# Resting-State Brain Abnormalities in Chronic Subjective Tinnitus: A Meta-Analysis

**DOI:** 10.3389/fnhum.2017.00022

**Published:** 2017-01-24

**Authors:** Yu-Chen Chen, Fang Wang, Jie Wang, Fan Bo, Wenqing Xia, Jian-Ping Gu, Xindao Yin

**Affiliations:** ^1^Department of Radiology, Nanjing First Hospital, Nanjing Medical UniversityNanjing, China; ^2^Department of Otolaryngology, Nanjing First Hospital, Nanjing Medical UniversityNanjing, China; ^3^Department of Endocrinology, Nanjing First Hospital, Nanjing Medical UniversityNanjing, China; ^4^Department of Vascular and Interventional Radiology, Nanjing First Hospital, Nanjing Medical UniversityNanjing, China

**Keywords:** tinnitus, neuroimaging, meta-analysis, resting-state fMRI, brain networks

## Abstract

**Purpose**: The neural mechanisms that give rise to the phantom sound of tinnitus have not been fully elucidated. Neuroimaging studies have revealed abnormalities in resting-state activity that could represent the neural signature of tinnitus, but there is considerable heterogeneity in the data. To address this issue, we conducted a meta-analysis of published neuroimaging studies aimed at identifying a common core of resting-state brain abnormalities in tinnitus patients.

**Methods**: A systematic search was conducted for whole-brain resting-state neuroimaging studies with SPECT, PET and functional MRI that compared chronic tinnitus patients with healthy controls. The authors searched PubMed, Science Direct, Web of Knowledge and Embase databases for neuroimaging studies on tinnitus published up to September 2016. From each study, coordinates were extracted from clusters with significant differences between tinnitus subjects and controls. Meta-analysis was performed using the activation likelihood estimation (ALE) method.

**Results**: Data were included from nine resting-state neuroimaging studies that reported a total of 51 distinct foci. The meta-analysis identified consistent regions of increased resting-state brain activity in tinnitus patients relative to controls that included, bilaterally, the insula, middle temporal gyrus (MTG), inferior frontal gyrus (IFG), parahippocampal gyrus, cerebellum posterior lobe and right superior frontal gyrus. Moreover, decreased brain activity was only observed in the left cuneus and right thalamus.

**Conclusions**: The current meta-analysis is, to our knowledge, the first to demonstrate a characteristic pattern of resting-state brain abnormalities that may serve as neuroimaging markers and contribute to the understanding of neuropathophysiological mechanisms for chronic tinnitus.

## Introduction

Tinnitus is the conscious perception of sound in the absence of an internal or external acoustic signal (Jastreboff, 1990). In the United States, an estimated 50 million adults have experienced tinnitus occasionally, and 16 million adults are estimated to experience frequent tinnitus (Shargorodsky et al., 2010). The central nervous system is believed to play a major role in its development and maintenance of tinnitus (Rauschecker et al., 2010; Leaver et al., 2011; De Ridder et al., 2014b; Chen et al., 2015a). Previous electrophysiological and neuroimaging studies (Lockwood et al., 1998; Kaltenbach et al., 2005) suggest that tinnitus may arise from aberrant firing patterns or high levels of spontaneous neural activity in the central auditory pathway rather than the cochlea. However, evidences suggest that other brain regions outside the classical auditory pathway may involve attentional mechanisms that contribute to the persistent awareness of the phantom sound as well as the development of anxiety and distress leading to disabling features of chronic tinnitus (Mirz et al., 2000; Schmidt et al., 2013; Henry et al., 2014). Despite extensive research, the brain abnormalities and neuropathophysiological mechanisms underlying chronic tinnitus remain poorly understood. One of the major questions is what regions of the human brain are involved in tinnitus, a question that has been explored in numerous imaging studies (Jastreboff et al., 1994; Lockwood et al., 1998; Mirz et al., 2000; Rauschecker et al., 2010; De Ridder et al., 2014b).

A number of existing neurophysiological mechanisms and models have been proposed to account for the pathophysiology of tinnitus patients, such as central gain (Schaette and McAlpine, 2011), neural synchrony (Seki and Eggermont, 2003), frontostriatal gating (Rauschecker et al., 2015), thalamocortical dysrhythmia (Llinás et al., 1999), noise-canceling deficit (Rauschecker et al., 2010; Leaver et al., 2011), global workspace (De Ridder et al., 2014b), and precision/predictive coding models (Sedley et al., 2016). However, there is a lack of consensus as to which neural mechanism(s) and what regions of the central nervous system are common to the diverse population of tinnitus patients participating in the imaging studies. Human neuroimaging studies have revealed augmented activity in tinnitus patients in several brain regions within and/or beyond classical auditory pathways. According to the model of De Ridder et al. (2014b) tinnitus is underpinned by the integration of multiple nonspecific subnetworks of the brain involving general components of cognition, emotion and memory. Communication between these different subnetworks occurs at specific hubs, brain areas that are involved in multiple subnetworks simultaneously. These different subnetworks, which interact at different oscillatory frequencies, communicate with one another at partially overlapping hubs. Nonetheless, the role of the potential hubs involved in multiple subnetworks of tinnitus still remains unclear.

Since about 85% of chronic tinnitus patients perceived the phantom sound constantly (Schecklmann et al., 2014), the resting-state fMRI measurements seem well suited to identify the neural structures, subnetworks and hubs involved in tinnitus. A growing number of studies have used resting-state fMRI to investigate tinnitus (Husain and Schmidt, 2014) and multiple brain networks implicated in tinnitus have been identified, such as the auditory network (Burton et al., 2012; Kim et al., 2012; Maudoux et al., 2012a,b; Schmidt et al., 2013; Hinkley et al., 2015; Minami et al., 2015; Leaver et al., 2016b), default mode network (DMN; Schmidt et al., 2013; Chen et al., 2014, 2015d; Leaver et al., 2016b), dorsal attention network (DAN; Burton et al., 2012; Schmidt et al., 2013), ventral attention network (VAN; Burton et al., 2012), and visual network (Burton et al., 2012; Chen et al., 2014, 2015d). As such, tinnitus can be seen as the interaction of multiple brain subnetworks, each contributing to different aspects of tinnitus such as its acoustic features, emotional affect and awareness or attention. However, these studies reported relatively inconsistent results. For instance, most researches demonstrated the increased resting-state brain activity between tinnitus patients and healthy controls (Maudoux et al., 2012a; Chen et al., 2014, 2016; Laureano et al., 2014; Yang et al., 2014; Ueyama et al., 2015), while others failed to identify any regions of increased brain activity (Geven et al., 2014; Leaver et al., 2016b). Moreover, several studies have failed to detect any differences in network processing between tinnitus patients and controls (Wineland et al., 2012; Davies et al., 2014). These reported discrepancies can potentially be attributed to the limited sample size, variable clinical demographics and use of different methods. Furthermore, in many studies tinnitus groups were not compared to an appropriate control group (Leaver et al., 2011; Schecklmann et al., 2013; Lanting et al., 2014).

Tinnitus is a very heterogeneous condition with respect to the characteristics of the perceived sound, degree of associated awareness and distress, duration and comorbidities (Landgrebe et al., 2012). Given the high heterogeneity, it is not surprising that inconsistencies across studies are encountered. Nevertheless, there may be some commonalities across these diverse studies. One approach to identifying a common core is to perform a meta-analysis of the existing resting-state studies of patients with chronic tinnitus with the goal of identifying brain network hubs in tinnitus patients common to neuroimaging studies of tinnitus. Activation likelihood estimation (ALE) is the most common coordinate-based meta-analytic method used to analyze the neuroimaging literature; this approach seeks to identify brain locations with a consistent pattern of response across experiments. This approach is based on the collection of peak coordinates from each study included in the meta-analysis rather than the input of raw images (Turkeltaub et al., 2002; Wager et al., 2004; Laird et al., 2005a; Eickhoff et al., 2009). ALE technique has been successfully applied to neuroimaging studies of various neurological or psychiatric disorders, such as epilepsy (Li et al., 2012), Parkinson’s disease (Shao et al., 2015), schizophrenia (Ellison-Wright and Bullmore, 2010), and narcolepsy (Weng et al., 2015). Song et al. (2012) performed a meta-analysis exclusively of PET studies of tinnitus using the ALE method to retrieve the most consistent activation areas across different task dimensions. However, this meta-analysis did not include the large body of fMRI studies that might prove useful in detecting resting-state brain abnormalities in tinnitus patients. Therefore the goal of the current study was to conduct a quantitative meta-analysis of several different types of resting state neuroimaging data in the tinnitus literature that met our inclusion criterion. To accomplish this, we used the ALE algorithm to determine the resting-state brain abnormalities in tinnitus patients compared to healthy controls. Our working hypothesis was that the ALE analysis would identify a common core of brain regions linked to tinnitus generation despite the heterogeneity of the patients and experimental methods.

## Materials and Methods

### Search Strategies and Study Selection

Our analysis was performed according to the Meta-Analysis of Observational Studies in Epidemiology (MOOSE) criteria (Stroup et al., 2000). A comprehensive literature search up to September, 2016 was conducted in PubMed, Science Direct, Web of Knowledge, and Embase using the following search terms: (1) “neuroimaging” <OR > “PET” <OR > “fMRI”; (2) “resting state”; and (3) “tinnitus”. Our search was restricted to humans. In addition, we manually reviewed the references cited in articles that were retrieved.

**Figure 1:**
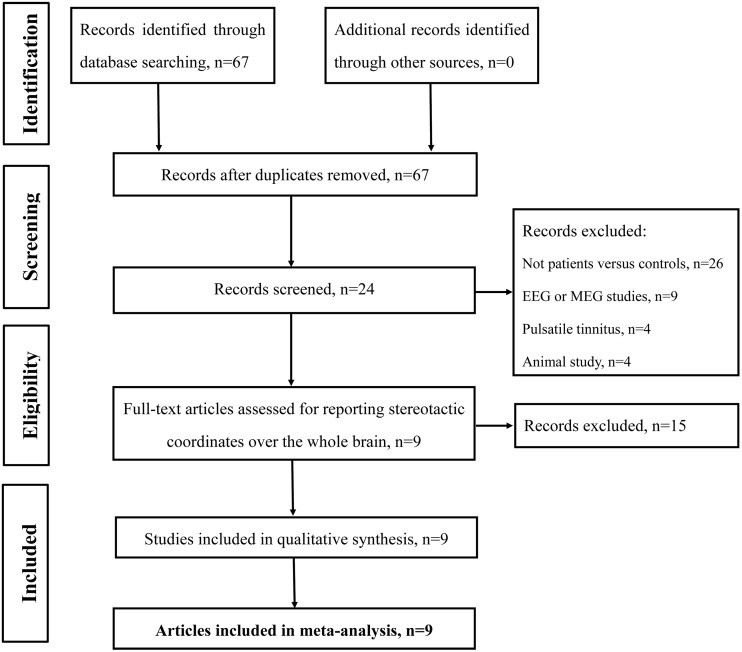
**Flow diagram of the literature search.** Flow diagram shows the results of the systematic search for the selected studies in this meta-analysis.

Studies were selected according to the following inclusion criteria: (1) published as an article (and not a letter or an abstract); (2) comparisons of tinnitus patients with healthy control groups on a whole-brain level; and (3) clearly reported Montreal Neurological Institute (MNI) or Talairach coordinates of the activation areas (*x*, *y*, *z*). Studies reporting only findings for specific ROIs were not included in the present meta-analysis. In accordance with many previous ALE meta-analyses (Laird et al., 2005b; Petacchi et al., 2005; Li et al., 2010; Kühn and Gallinat, 2013), we included coordinates resulting from fMRI as well as from PET data. We included data from PET and fMRI studies and other data analysis techniques despite the fact that they have different physiological bases and theoretical assumptions since both methods have been used to identify differences between the intrinsic brain function in patients compared with controls. The rationale was to provide an all-encompassing overview of attempts to identify resting-state abnormalities in tinnitus patients. Our search criteria yielded a total of 67 peer-reviewed published articles. Of the 67 studies, 26 studies were excluded as these studies were not about comparisons between tinnitus patients and healthy controls; nine articles were excluded since these were electroencephalography (EEG) or magnetoencephalography (MEG) studies; another four pulsatile tinnitus studies and four animal studies were excluded. Fifteen studies were further excluded since these did not report detailed peak coordinates over the whole brain. Finally, nine resting-state neuroimaging studies, six fMRI, two SPECT and one PET, were included in the ALE meta-analysis (Figure 1 and Table 1).

**Table 1:** **List of all studies included in the meta-analysis: subjects’ demographic and clinical characteristics**.

Study	Journal	Modality/ Method of analysis	Male: Female		Mean age ± SD		Reported contrasts	Foci NO.	Scanner	Processing software	Smoothing kernel (mm)	Statistical threshold	MNI or Tal
			Patients	Control	Patients	Control
Maudoux et al. (2012a)	Plos One	fMRI/ICA	7:6	9:6	52 ± 11	51 ± 13	TIN > HC HC > TIN	11 6	Siemens 3.0T	Brain Voyager	8	*P* < 0.05, FDR corrected	Tal
Geven et al. (2014)	Neuroscience	PET	10:10	9:10	51.0 ± 10.0	50.8 ± 9.5	HC > TIN	2	Siemens	SPM5	8	*p* < 0.001, uncorrected	MNI
Laureano et al. (2014)	Plos One	SPECT	6:14	6:11	42.95 ± 9.03	41.41 ± 9.98	TIN > HC	1	GE	SPM8	8	*P* < 0.05, FWE corrected	MNI
Chen et al. (2014)	NeuroImage: Clinical	fMRI/ALFF	17:14	17:15	41.9 ± 10.8	46.5 ± 12.6	TIN > HC HC > TIN	3 4	Siemens 3.0T	SPM8	4	*P* < 0.05, AlphaSim corrected	MNI
Yang et al. (2014)*	Journal of Otology	fMRI/ReHo	14:4	15:5	43	42	TIN > HC HC > TIN	1 1	Philips3.0T	SPM5	NA	*P* < 0.05, FWE corrected	MNI
Ueyama et al. (2015)	Plos One	SPECT	10:7	NA	NA	NA	TIN > HC HC > TIN	6 2	FUJI FILM	SPM8	8	*P* < 0.05, AlphaSim corrected	MNI
Chen et al. (2015d)	Neural Plasticity	fMRI/ReHo	16:13	15:15	40.9 ± 10.5	46.2 ± 11.9	TIN > HC HC > TIN	4 1	Siemens 3.0T	SPM8	4	*P* < 0.01, AlphaSim corrected	MNI
Leaver et al. (2016b)	Human Brain Mapping	fMRI/ICA	10:11	9:10	47.33 ± 13.47	48.89 ± 12.49	HC > TIN	5	Siemens 3.0T	Brain Voyager	6	*P* < 0.0005, uncorrected	Tal
Chen et al. (2016)	Frontiers in Aging Neuroscience	fMRI/DC	9:15	9:13	50.8 ± 12.4	44.7 ± 15.4	TIN > HC	2	Philips 3.0T	SPM8	6	*P* < 0.01, AlphaSim corrected	MNI

Note: TIN, tinnitus; HC, healthy control; fMRI, functional magnetic resonance imaging; PET, positron emission tomography; SPECT, single photon emission computed tomography; ALFF, amplitude of low-frequency fluctuations; ReHo, regional homogeneity; ICA, independent component analysis; DC, degree centrality; SPM, statistical parametric mapping; MNI, Montreal Neurological Institute; FDR, false discovery rate; FWE, family-wise error; NA, not available. *Only reported mean age in this study.

Two independent reviewers (CYC and FW) evaluated the methodology and the risk of bias of the eligible studies. First, they assessed the titles of the search results and retrieved the relevant articles. Second, the articles that remained eligible were assessed based on their abstract to determine whether any of the inclusion criteria were not met. The full text of all remaining articles was then assessed with a data extraction template, which was constructed for the purpose of organizing and extracting information from the included articles and excluding articles without peak values. The reviewers analyzed all articles in terms of patient selection and their comparable controls, blinding, diagnostic criteria and regression methods. Any disagreements were assessed by the third reviewer (FB). The demographic data were extracted from each article, including the first author’s name, year of publication, total patient number, sex distribution, mean patient age and range, statistic thresholds, hearing and psychological status.

### Data Extraction

The *x*, *y*, and *z* peak activation coordinates of all eligible contrasts constituted the meta-analysis input. The data originally reported in Talairach spaces were converted to MNI coordinates (Lancaster et al., 2007). The data from MNI coordinates were texted and implemented in GingerALE 2.3.3^1^, Research Imaging Institute of the University of Texas Health Science Center, San Antonio, TX, USA). Coordinates in each study were independently extracted by two authors (CYC and FW).

### ALE Meta-Analysis

Ginger ALE software was used to analyze the resting-state brain activity between tinnitus patients and healthy controls. The reported loci of maximal anatomical differences were modeled as the peaks of three-dimensional Gaussian probability density functions defined by the full-width at half-maximum (FWHM), which was set according to a quantitative uncertainty model (Laird et al., 2005a; Eickhoff et al., 2009). ALE values were calculated on a voxel-by-voxel basis by measuring the union model activation maps modeled above. This revised analysis tested for convergence by studies (random effects) instead of foci (fixed effects). Following the method described by Turkeltaub et al. (2002, 2012), 1000 permutations were used to determine which tests were statistically significant and threshold determined for the resultant ALE map. A whole-brain histogram was computed in which the null hypothesis of uniformly distributed foci was rejected for voxels with an ALE value greater than the critical threshold, defined as the 100(1−α)th percentile of the permutation distribution, where *α* refers to the desired level of significance. The analyses were performed at a cluster forming threshold (reported with each *p* value and ALE thresholds in the results, ALE values greater than this threshold are statistically significant) computed using a *p* < 0.05, corrected for multiple comparisons using false-discovery rate (FDR; Genovese et al., 2002; Laird et al., 2005a). The volume of the minimum cluster threshold was set at 200 mm^3^. The coordinates of the weighted center were generated for each cluster. The resulting significant anatomical areas were labeled based on probabilistic cytoarchitectonic maps of the human brain using the SPM Anatomy Toolbox v2.1 (Eickhoff et al., 2005). Results were visualized with Mango software^2^, using the Colin brain template in the MNI space^3^.

## Results

Using our inclusion/exclusion criteria, we identified nine eligible neuroimaging studies utilizing different methods, including SPECT (Laureano et al., 2014; Ueyama et al., 2015), PET (Geven et al., 2014), and fMRI (Maudoux et al., 2012a; Chen et al., 2014, 2015d, 2016; Leaver et al., 2016b). Figure 1 is a flow diagram showing the steps in the identification, exclusion and inclusion of the studies. The clinical and demographic data of the participants from all recruited studies are presented in Table 1. The subjects of patients and controls from each study are generally comparable by age, sex, and education. The hearing and psychological status are also shown in Table 2.

**Table 2:** **The hearing and psychological status of the subjects included in all the studies**.

Study	Hearing status		Psychological status
	Patients	Controls
Maudoux et al. (2012a)	2 severe HL	No HL	No major neurological neurosurgical or psychiatric history
	7 mild/moderate HL
	4 no HL
Geven et al. (2014)	NA	NA	No major medical, neurological or psychiatric diagnoses
Laureano et al. (2014)	No HL	No HL	No neurologic or psychiatric disorders
Chen et al. (2014)	No HL	No HL	No depression or anxiety or other neurologic or psychiatric disorders
Yang et al. (2014)	1 profound HL	No HL	NA
	1 severe HL
	4 moderate HL
	9 mild HL
	3 no HL
Ueyama et al. (2015)	11 mild to severe HL	No HL	No neurologic or psychiatric disorders
	6 no HL
Chen et al. (2015d)	No HL	No HL	No depression or anxiety or other neurologic or psychiatric disorders
Leaver et al. (2016b)	15 mild to severe HL	10 mild to severe HL	No significant symptoms of anxiety or depression
	6 no HL	9 no HL	
Chen et al. (2016)	No HL	No HL	No depression or anxiety or other neurologic or psychiatric disorders

*Note: HL, hearing loss; NA, not available*.

**Figure 2:**
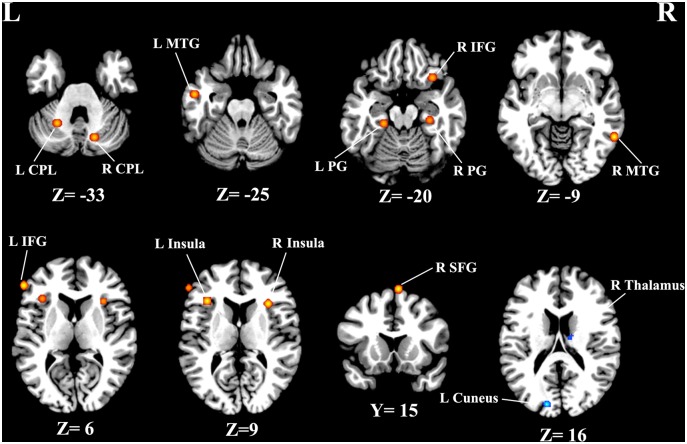
**Resting-state brain activity alterations in chronic tinnitus patients compared with healthy controls.** Results are from the activation likelihood estimation (ALE) software for meta-analyses. All activations are significant at *p* < 0.05 corrected for multiple comparisons using the false-discovery rate (FDR) correction.

As illustrated in Figure 2, a total of 13 peak foci were reported in this meta-analysis. Compared with healthy controls, tinnitus patients showed increased resting-state neural activity bilaterally in the middle temporal gyrus (MTG), inferior frontal gyrus (IFG), parahippocampal gyrus, insula, cerebellar posterior lobe and right superior frontal gyrus (SFG). Moreover, decreased brain activity was also observed in the left cuneus and right thalamus. Table 3 displays the coordinates of cluster maxima.

**Table 3:** **Regions of altered brain activity in tinnitus patients relative to healthy controls**.

Brain regions	BA	MNI coordinates *x, y, z* (mm)	ALE extrema value	Cluster size (mm^3^)
**Tinnitus > Controls**
L Insula	13	−36, 24, 9	0.0088	880
R Cerebellar Posterior Lobe	–	17, −72, −33	0.0075	768
L Cerebellar Posterior Lobe	–	−25, −56, −33	0.0075	768
L Middle Temporal Gyrus	21	−53, −6, −25	0.0075	768
R Middle Temporal Gyrus	37	60, −51, −9	0.0086	768
R Inferior Frontal Gyrus	47	34, 22, −20	0.0072	736
R Superior Frontal Gyrus	6	6, 15, 69	0.0086	728
L Parahippocampal Gyrus	35	−22, −30, −20	0.0073	720
R Parahippocampal Gyrus	35	30, −26, −18	0.0073	720
L Inferior Frontal Gyrus	46	−57, 39, 6	0.0085	712
R Insula	13	34, 20, 10	0.0082	704
**Tinnitus < Controls**
L Cuneus	17	−14, −88, 16	0.013	488
R Thalamus	–	14, −10, 14	0.0101	368

*ALE, activation likelihood estimation; BA, Brodmann area; MNI, Montreal Neurological Institute; L, left; R, right. Each ALE map was thresholded using a FDR-corrected p < 0.05*.

## Discussion

The current study is the first whole-brain meta-analysis exploring the resting-state brain abnormalities in chronic tinnitus patients compared to healthy controls. By analyzing nine neuroimaging studies, the meta-analysis identified consistent regions of aberrant neural activity mainly in the non-auditory brain regions, including the MTG, frontal cortex, parahippocampus, insula, cerebellum, cuneus, and thalamus, in different aspects of tinnitus. Surprisingly, the auditory cortex was not detected in this study. This may be due to the fact that most patients in these studies had little or mild hearing loss. These non-auditory areas are parts of separable subnetworks representing multiple clinical cognitive and emotional aspects of tinnitus; resting-state disruptions in these areas may provide new insights on the neuropathological mechanisms of this disorder.

### Middle Temporal Gyrus

The MTG has been suggested to be involved in cognitive processes including language, semantic memory and multimodal sensory integration (Cabeza and Nyberg, 2000). A quantitative EEG study has demonstrated higher α2-band activity in the MTG in tinnitus patients with higher distress (Vanneste et al., 2010a). Voxel-based morphometry (VBM) analyses revealed that the gray matter increases in the MTG in tinnitus patients with hearing impairment (Boyen et al., 2013). Using resting-state fMRI, significantly increased spontaneous neural activity was observed in the right MTG in tinnitus patients (Chen et al., 2014). Moreover, Zhang et al. (2015) showed decreased functional connectivity between right MTG and left thalamus, which was negatively correlated with tinnitus severity. In addition, the MTG has been regarded as a key region of the DMN (Raichle et al., 2001). The DMN, consisting of nodes in the MTG, posterior cingulate/precuneus, angular gyrus and medial frontal gyrus, is most active at rest and shows reduced activity when a subject enters a task-based state involving attention or goal-directed behavior (Raichle et al., 2001; Mantini et al., 2007). As a condition involving the perception of a phantom auditory sensation, tinnitus might lead to dysfunction of the DMN. Previous fMRI studies found abnormal functional connectivity within the DMN associated with tinnitus distress (Burton et al., 2012; Maudoux et al., 2012b; Schmidt et al., 2013). Nevertheless, the source or type of aberrant neural activity within DMN regions in tinnitus remains unclear. Our results indicate that increased neuronal activity in the MTG may be responsible for disrupting the DMN in tinnitus patients.

### Frontal Cortex

The frontal cortex, including the SFG and IFG, exhibited increased neural activity in tinnitus patients in the current study. Rauschecker et al. (2010) developed a model to demonstrate structural and functional differences in ventromedial prefrontal cortex that were associated with tinnitus subjective loudness, indicating that frontal cortex may contribute to certain perceptual features of tinnitus. Resting-state fMRI studies have pointed out that the abnormalities of the frontal cortex could act as a direct mechanism of tinnitus chronification (Burton et al., 2012; Schmidt et al., 2013; Chen et al., 2014, 2015c,d, 2016), which are confirmed by the current meta-analysis. Based on the previous fMRI studies, the SFG has been regarded as a major integrative hub of the tinnitus network architecture (Chen et al., 2016), which can receive and integrate all kinds of information from different parts of the brain from inside and outside the body. Besides, it can also timely organize efferent impulses to ensure the coordination of the central nervous system as a whole (Mathiak et al., 2007; Melloni et al., 2007). A possible explanation for our result was that the increased activity of the SFG might be due to feedback inhibition of an over active auditory network (Downar et al., 2000). Furthermore, the IFG serves as the core region of response inhibition and IFG activity might mirror the attempt to control the bottom-up attention allocation to the tinnitus percept in a top-down manner (Aron et al., 2014). In one hypothetical model, the fronto-insular cortex is part of a salience network that drives switching by a central executive control network important to maintaining and adjusting attention (Sridharan et al., 2008). In another model, the IFG and the insula act as executive control components in the attention system that regulates dorsal and VANs, which lack direct interconnections (Shulman et al., 2009). Taken together, we suggest that tinnitus distress, salience, or attentional focus is associated with increased resting-state activity in these brain subnetworks.

### Parahippocampus

The parahippocampal area has been hypothesized to play a central role in memory recollection and transferring information from the hippocampus to the association areas, which might explain its involvement in the generation of simple auditory phantom percepts such as tinnitus (De Ridder et al., 2014a; Vanneste and De Ridder, 2016). EEG study suggested that tinnitus patients differed from healthy controls by increased delta and theta activity in the parahippocampus (Moazami-Goudarzi et al., 2010). Prior resting-state fMRI studies also provided further support linking tinnitus physiopathology with parahippocampal region involved in mnemonic network (Maudoux et al., 2012a; Chen et al., 2015b; Leaver et al., 2016b).

### Insula

The increased response in the insula, mainly the anterior part, may be an indication of successful adaption to the tinnitus perception (van der Loo et al., 2011). On the basis of resting-state quantitative EEG, the insula has been implicated in tinnitus and specific tinnitus characteristics (van der Loo et al., 2011; Vanneste et al., 2011a,b). Greater synchrony of alpha activity was observed bilaterally in the anterior insula of patients with more severe tinnitus-related distress (Vanneste et al., 2010a). In addition, chronic tinnitus patients showed enhanced spontaneous neuronal activity and functional connectivity in bilateral anterior insula were revealed by resting-state fMRI (Burton et al., 2012; Chen et al., 2015d).

Furthermore, the insula is one of key nodes in the common brain circuit of both tinnitus and chronic pain (Rauschecker et al., 2015). Although there also exist important differences between the two disorders, the striking similarities and associations indicate that tinnitus and chronic pain share common neuropathological mechanisms. Now advances in neuroimaging have demonstrated that similar structures and functional systems are involved and probably play a central role in both disorders (Rauschecker et al., 2015). Significant loss of gray matter and compromised circuit function are detected in several specific regions, such as the ventromedial prefrontal cortex, nucleus accumbens and insula (De Ridder et al., 2011; Rauschecker et al., 2015). These areas act as a central gatekeeping system for perceptual sensations, which determines the affective value of sensory stimuli and modulates information flow in the brain (Rauschecker et al., 2015). Tinnitus and chronic pain occur when this system is compromised.

Although the EEG has been used to determine resting-state long-range functional coupling in tinnitus, it has many differences from the resting-state fMRI (Vanneste et al., 2010a,b). EEG does not offer the spatial resolution of fMRI, but offer the advantage of being quiet and not influencing the resting-state network of tinnitus. Thus these two techniques may measure different aspects of spontaneous neuronal activity (Tagliazucchi et al., 2012). Britz et al. (2010) extracted four resting-state networks from the EEG data that were the equivalent of stereotypical fMRI networks dedicated to auditory, attetional, visual and self-referential processing, but DMN was not detected by EEG in Britz’s study. Other studies have correlated the DMN with beta-2 (Laufs et al., 2003) or with delta (Mantini et al., 2007) spectral bands of EEG. Therefore, a direct comparison of EEG and resting-state fMRI is complicated by the fact that similar EEG power bands may be correlated with varying fMRI-generated spatial maps and a single resting-state network may be linked with different EEG spectral patterns (Laufs et al., 2008; Musso et al., 2010).

### Cerebellum

Furthermore, although the cerebellum is primarily involved in motor actions and control, several cerebellar regions such as the paraflocculus and vermis receive inputs from auditory centers (Petacchi et al., 2005). Brozoski et al. (2007) observed increased activity in the parafloccular lobe of the cerebellum in animals with tinnitus confirmed and suggested that the cerebellum acts as a gating control mechanism comparing the afferent input from the cochlea with descending signals from the auditory cortex (Bauer et al., 2013; Chen et al., 2015b). Consistent with this view, hyperactivity in the auditory cortex and increased functional connectivity between the auditory cortex and cerebellum were observed in rats with salicylate-induced tinnitus (Chen et al., 2015a). If this cerebellar-tinnitus gating hypothesis is correct, then inactivating the cerebellum could possibly suppress the sound perception of tinnitus. Based on these findings, we suggest that cerebellum may play a pivotal role of gating control in tinnitus. Furthermore, using resting-state fMRI, chronic tinnitus patients exhibited enhanced functional connectivity in the cerebellar hemisphere that was associated with tinnitus distress (Maudoux et al., 2012a; Ueyama et al., 2013), indicating the involvement of cerebellum in auditory system.

### Cuneus

Our meta-analysis found decreased brain activity in the cuneus of tinnitus patients. We speculate that the connections between auditory and visual regions make it possible to alter the brain activity in the visual areas (Kaltenbach et al., 2004; Cate et al., 2009). This is consistent with prior fMRI studies showing negative correlations of functional connectivity between auditory and visual resting-state subnetworks in tinnitus patients (Burton et al., 2012; Maudoux et al., 2012b). One possibility is that the phantom sounds might act to decrease spontaneous activity in visual areas because of the salience of the tinnitus perception (Chen et al., 2014, 2015d). Thus, tinnitus may be regarded as the consequence of multisensory interactions between auditory and visual regions.

### Thalamus

The thalamus, which regulates the flow of sensory information to and from the auditory cortex, has been thought to play a key role in tinnitus (Richardson et al., 2012). Llinás et al. (1999) hypothesized that tinnitus results from thalamocortical dysrhythmias triggered by peripheral damage. Prior MRI studies have identified structural and functional abnormalities involved in thalamocortical network of tinnitus (Mühlau et al., 2006; Rauschecker et al., 2010; Benson et al., 2014; Chen et al., 2014; Lanting et al., 2014; Zhang et al., 2015). Consistent with our results, Zhang et al. (2015) showed decreased functional connectivity between the thalamus and auditory cortical areas in tinnitus, which may be a reflection of disrupted thalamic gating mechanism (Rauschecker et al., 2010). The thalamus is regarded as the center of ascending noise canceling system, and thus if it is dysfunctional, noise canceling is no longer possible and the subject may perceive tinnitus (Rauschecker et al., 2010). However, the mechanisms responsible for these functional changes are unknown, but could involve aberrant inhibition (Richardson et al., 2012).

### Limitations

Several inevitable limitations should be noted in our study. First, the heterogeneity of the included studies could affect the current results. In particular, hearing loss is always a complicating factor in structural and functional studies of tinnitus. Melcher et al. (2013) compared tinnitus patients with controls who were matched for hearing thresholds in the standard clinical frequencies and found that gray matter changes were not related to tinnitus but instead negatively correlated with hearing thresholds at frequencies above 8 kHz. Therefore, a more complete characterization of hearing loss is desirable. Moreover, tinnitus can become a significant psychological problem and comorbid symptoms such as depression and anxiety are potential confounds on the observed brain activity in tinnitus patients (Leaver et al., 2016a). It is necessary to characterize the psychological health problems as covariates in the analyses. The problems of differential tinnitus variables on structural and functional changes in neural activity can be solved using correlational analyses of large samples (Schecklmann et al., 2013) and comparisons of different tinnitus subtypes (Vanneste and De Ridder, 2012). Second, voxel-wise meta-analytic approach like ALE provides excellent control of false positive results, but it is more difficult to avoid false negatives (Radua et al., 2012). Voxel-wise meta-analysis is based on the pooling of peak stereotactic coordinates rather than on raw statistical brain maps from the original studies, which could give rise to less accurate results (Radua et al., 2012). Furthermore, fMRI produces aversive scanner noise which has been shown to interfere with auditory processing in the human brain at the physiological and psychological level (Perrachione and Ghosh, 2013). The concept of resting state is somewhat problematic in tinnitus studies because the auditory pathway is likely to be activated by scanner noise which is nearly impossible to completely eliminate even with ear plugs or active noise reduction (Logothetis et al., 2009). Therefore, this limitation should be taken into consideration when interpreting the resting-state fMRI data in auditory system-related researches. Finally, the number of included neuroimaging studies is relatively small. Further explorations are needed to perform the subgroup analyses or meta-regression analyses for the control of confounding factors that might influence resting-state brain function in tinnitus, such as the imaging methodology and data analysis method. Most importantly, we should establish standard protocols for resting-state fMRI researches to minimize heterogeneity on the experimental side.

## Conclusion

Using the ALE-based meta-analysis, our study demonstrated abnormal resting-state neural activity mainly in the non-auditory brain areas, including the MTG, frontal cortex, parahippocampus, insula, cerebellum, cuneus and thalamus. In accordance to the hypothesis, these results show that there exist several important brain hubs in specific tinnitus network encompassing DMN, attentional, mnemonic, salience, visual and thalamocortical subnetworks. Aberrant resting-state brain patterns in tinnitus-related networks may help enhance our understanding of the neuropathological mechanisms underlying chronic subjective tinnitus. Nonetheless, a theoretical pathophysiological framework capable of explaining all these aspects in one model is still highly required. There is thus hope that a single cure can be found that would target a common mechanism.

## Author Contributions

Y-CC and FW designed the experiment, reviewed the literatures, performed the analysis and wrote the manuscript. JW, FB and WX reviewed the literatures. J-PG and XY contributed to the discussion and manuscript revision.

## Conflict of Interest Statement

The authors declare that the research was conducted in the absence of any commercial or financial relationships that could be construed as a potential conflict of interest. The reviewer MM and handling Editor declared their shared affiliation, and the handling Editor states that the process nevertheless met the standards of a fair and objective review.

## Footnotes

^1^http://brainmap.org/ale/

^2^http://ric.uthscsa.edu/mango/mango.html

^3^http://www.brainmap.org/ale
